# Protein kinase C-α (PKCα) modulates cell apoptosis by stimulating nuclear translocation of NF-kappa-B p65 in urothelial cell carcinoma of the bladder

**DOI:** 10.1186/s12885-017-3401-7

**Published:** 2017-06-19

**Authors:** Jin Zheng, Chuize Kong, Xiaoxi Yang, Xiaolu Cui, Xuyong Lin, Zhe Zhang

**Affiliations:** 1 Department of Urology, The First Affiliated Hospital of China Medical University, Shenyang, Liaoning 110001 China; 2 Department of Cardiovascular, The First Affiliated Hospital of China Medical University, Shenyang, Liaoning 110001 China; 3 Department of Pathology, The First Affiliated Hospital and College of Basic Medical Sciences, China Medical University, Shenyang, Liaoning 110001 China

**Keywords:** PKCα, NF-κB, Urothelial cell cancer, Apoptosis

## Abstract

**Background:**

The protein kinase C (PKC) family comprises central regulators of multiple signal transduction processes and is involved in the progression of many cancers. Nuclear factor Kappa-B (NF-κB) is constitutively expressed in cancer tissues and stimulates the transcription of various tumor-related genes. The present study aims to investigate the clinical significance of PKCα and NF-κB p65 in bladder cancer tissues and the mechanism underlying PKCα induction of bladder cancer cell apoptotic resistance through stimulation of p65 nuclear translocation.

**Methods:**

Expression of PKCα and NF-κB subunit p65 was detected in seven bladder cancer cell lines by western blot and in 30 bladder cancer tissue specimens by immunostaining. Immunofluorescence was performed to evaluate p65 nuclear translocation induced by Phorbol 12-myristate 13-acetate (PMA). PKCα/β selective inhibitor Gö6976, PKC pan-inhibitor sotrastaurin, and the PKC siRNA were employed to conduct PKC inhibition/knockdown in bladder cancer cells. Luciferase reporter assays were performed to measure the activity of NF-κB. Flow cytometry and TUNEL analysis were used to assess cell apoptosis.

**Results:**

Expression of PKCα and NF-κB was found to positively correlate with tumor progression in 30 tumor tissue specimens. Furthermore, a Pearson’s correlation coefficient analysis revealed a positive correlation between PKCα and NF-κB expression. Among the PKC inhibitors, the PKCα/β selective inhibitor Gö6976 yielded the most significant block of PKCα and NF-κB activation by PMA. Knockdown of NF-κB p65 remarkably induced cell apoptosis, but PMA restored p65 expression and significantly suppressed cell apoptosis that was otherwise induced by the p65 knockdown alone.

**Conclusion:**

Our study showed that PKCα modulated cell resistance to apoptosis by stimulating NF-κB activation and thus promoted the tumorigenesis of bladder cancer.

**Electronic supplementary material:**

The online version of this article (doi:10.1186/s12885-017-3401-7) contains supplementary material, which is available to authorized users.

## Background

Cancer is a major disease burden and public health problem globally [1]. Among the cancer types, bladder cancer is the ninth most common cancer worldwide [1] and the sixth most diagnosed cancer in China [2, 3]. Among the bladder cancers, more than 90% of the cases are urothelial cell carcinomas (UCCs). The main problems for bladder cancers are the high recurrence rate (50–70% of newly diagnosed superficial tumors will recur [4]) and the high progression rate (10–20% of superficial tumors will eventually progress to muscle invasive disease [5]). Thus, predicting patient outcomes and preventing disease progression remain big challenges.

Protein kinase C (PKC) is a family of serine/threonine kinases that regulates a variety of cellular biological process, such as cell motility, differentiation, survival and apoptosis [6–8]. The PKC family is classified into three groups: conventional PKCs (cPKCs, including PKCα, β and γ), novel PKCs (nPKCs, including PKCδ, ε, η and θ) and atypical PKCs (aPKCs, PKCζ and ι). It has been firmly established that PKCs are closely related to the process of tumorigenesis, including the initiation and progression of bladder cancer [9–11]. PKCα, a conventional PKC isoform, has been reported to be involved in the recurrence of bladder cancer [12]. Moreover, the expression pattern of PKCα in bladder carcinoma tissues is found to increase with tumor grade progression [9], which further indicates a tumorigenic role for PKCα in UCC of the bladder.

Nuclear factor kappa-B (NF-κB) is a family of transcription factors and has been widely recognized as a major determinant of the carcinogenesis of various human cancers [13–15]. Under resting conditions, NF-κB is localized to the cytoplasm and mainly exists as heterodimers of p50 and p65 [16]. In response to various extracellular stimuli such as cytokines, oxidative stress and cell damage, the inhibitory protein IκB, which is bound to the p65 subunit, is phosphorylated by IκB kinase (IKK) [17]. This permits nuclear translocation of NF-κB, which enhances the transcription of a wide variety of target genes [18]. PKC isozymes have been linked to the activation of NF-κB. PKC θ activates NF-κB through phosphorylation of the membrane-associated guanylate kinase (MAGUK) CARMA1 and regulates T cell function [19]. In breast cancer, PKC ζ is responsible for the activation of AP-1 and NF-κB [20]. PKCα has been reported to be associated with NF-κB activation in human lung epithelial cells [21]. To date, no systematic studies have investigated the mechanism of PKC activation of NF-κB signaling in UCC of the bladder.

Previous studies have noted that in bladder cancer, PKCα and NF-κB have similar effects or may cooperate in regulating cellular functions [10, 22, 23], which indicates that there may be underlying regulatory connections between these two factors. In the present study, we demonstrated that in UCC of the bladder, PKCα played a crucial role in regulating cell survival by stimulating the nuclear translocation of NF-κB subunit p65 with subsequent activation of NF-κB signaling. Our finding provides novel evidence to support the tumorigenic role of PKCα in bladder cancer tumorigenesis.

## Methods

### Tissue specimens and patient information

For the use of clinical materials for research purposes, prior patient written consent and approval were obtained from the China Medical University and The First Affiliated Hospital of China Medical University. A total of 30 patients with bladder urothelial cell carcinomas (BUCCs) underwent partial cystectomies and radical cystectomies from 2013 to 2015 at the Department of Urology of the First Affiliated Hospital of China Medical University (Table 1). Of these cases, 15 were pathologically diagnosed as BUCC with pT1 stage, and the other 15 were diagnosed as BUCC with pT4 stage. Histologically, the tumors were classified according to the 2004 World Health Organization histologic classification of urinary tract tumors and were staged using the 2002 American Joint Committee on Cancer system. The pathological sections of 30 BUCC tissue specimens were provided by the Department of Pathology at the First hospital of China Medical University, and the pathological diagnosis and analysis in this study were performed in collaboration with Department of Pathology.

**Table 1:** Association of PKCα and NF-κB p65 expression with clinicopathologic characteristics of the bladder cancer patients

Parameters	Group	No. of cases	*P*-value	
			PKCα	NF-κB p65
Gender	Male	17 (56.7%)	0.214	0.453
Age (years)	≥60	19 (63.3%)	0.371	0.284
Histological grade	High grade	21 (70%)	< 0.01**	< 0.01**
Muscle invasion	Positive	20 (66.7%)	<0.01**	<0.01**
Distant metastases	Positive	4 (13.3%)	0.124	0.073
Lymphatic invasion	Positive	6 (20%)	<0.05*	< 0.05*

PKCα and p65 expressions were measured by IHC staining, PKCα or p65 positive cell percentages per HPF were counted and statistically compared between two groups. Student’s T test was used to conduct the statistical analysis. *P < 0.05, **P < 0.01

### Cells and culture conditions

The human bladder carcinoma cell lines (T24, 5637, J82, RT4, UM-UC-3, and SW-780) and immortalized ureter epithelial cell line (SV-HUC-1) were purchased from the cell bank of Chinese Academy of Sciences (Shanghai, China). The respective catalog numbers for each cell line are as following: TCHu 55, TCHu 1, TCHu218, TCHu226, TCHu217, TCHu219 and TCHu169. The human bladder carcinoma cell line BIU-87 was obtained from the lab of oncology of our hospital as a gift. The cells were cultured in RPMI 1640 (HyClone, Logan, UT, USA) supplemented with 10% FBS (HyClone) and 1% penicillin-streptomycin (HyClone) at 37 °C under a humidified atmosphere with 5% CO_2_.

### RNA extraction and real-time quantitative PCR

Total RNA was extracted from cultured cell lines using the TRIzol reagent (Invitrogen) and reverse transcribed with random primers using the PrimeScript™ RT Master Mix (Perfect Real Time; Takara Biotechnology Co. Ltd., Dalian, China) according to the manufacturer’s instructions. qRT-PCR was performed to detect the levels of PKCs and β-actin using SYBR® Premix Ex Taq™ (Tli RNaseH Plus; Takara Biotechnology CO. LTD., Dalian, China) and the LightCycler™ 480 II system (Roche, Basel, Switzerland). β-actin was used as the internal control for each gene. The primer sequences are listed in Additional file 1: Table S1.

The relative levels of expression were quantified and analyzed using the LightCycler™ 480 software 1.5.1.6.2 (Roche, Basel, Switzerland). The real-time value for each sample was averaged and compared using the Ct method. The relative expression level (defined as the fold change) of each target gene (2^-ΔΔCt^) was normalized to the endogenous β-actin reference (ΔCt) and compared to the amount of the target gene in the control sample, which was calibrated to 1.0. Three independent experiments were performed to analyze the relative gene expression, and each sample was tested in triplicate.

### Protein extraction and western blotting

Cells were harvested in RIPA lysis buffer (Beyotime, Shenzhen, Guangdong, China) and boiled for 10 min at 90 °C. Protein concentrations were measured using the BCA assay. Approximately, 50 μg of the protein extract from cultured cells or 100 μg from fresh surgical bladder tissues were separated by 10% SDS-polyacrylamide gel electrophoresis (SDS-PAGE). The gels were then electrotransferred onto polyvinylidene difluoride (PVDF) membranes (Millipore, Billerica, MA, USA), which were then incubated with the indicated primary antibodies in 5% nonfat milk in TBS-T overnight at 4 °C. Next, the membranes were washed for 15 min and immediately incubated with anti-rabbit or anti-mouse horseradish peroxidase-conjugated secondary antibodies for 1 h at 37 °C. The housekeeping protein α-Tubulin (Sigma-Aldrich, St. Louis, MO, USA) was used as an internal control for the total protein measurement, and Histone H3 (Abcam, Cambridge, MA, USA) was used as a nucleoprotein reference. The bands were visualized using ECL reagents (Transgen Biotechnology, Beijing, China) on a MicroChemi Chemiluminescent Imaging System (DNR Bio-Imaging Systems, Mahale HaHamisha, Jerusalem, Israel). The densitometric values were calculated using the ImageJ 1.46r software (Wayne Rasband, National Institutes of Health, Bethesda, MA, USA), and the ratios of the target protein to α-tubulin/Histone H3 were used to conduct the statistical analysis.

### Nuclear/cytoplasmic fractionation

The Nuclear and Cytoplasmic Protein Extraction Kit (Beyotime, Shenzhen, Guangdong, China) was used to extract the nuclear and cytoplasmic proteins from cultured cells and tissues, according to the manufacturer’s protocol. Briefly, cells were washed with cold phosphate buffered saline (PBS), resuspended in buffer containing 1 mM DTT and 1 mM PMSF, and incubated on ice for 15 min. Detergent was added, and the cells were vortexed for 30 s at the highest speed. The nuclei and supernatant (cytoplasm) were separated by centrifugation at 4 °C. The nuclei were resuspended in buffer containing 1 mM DTT and 1 mM PMSF, incubated on ice for 30 min, and vortexed with interruptions. Nuclear extracts were collected by centrifugation at 14,000×*g* for 10 min at 4 °C. For nuclear protein extraction of tissues, 60 mg of frozen bladder tissues were excised, immediately suspended in buffer containing 1 mM DTT and 1 mM PMSF, homogenized on ice, and then incubated for 15 min. The subsequent procedure was the same as that for the cell nuclear and cytoplasmic protein extraction.

### Antibodies and reagents

Rabbit monoclonal antibody against PKCα (Phospho T638) (1:500 dilution) and rabbit polyclonal antibodies against PKCα (1:2000 dilution), NF-κB p65 (1:2000 dilution), and Histone H3 (1:3000 dilution) were purchased from Abcam (Cambridge, MA, USA). The rabbit polyclonal antibody against α-Tubulin (1:5000 dilution) was purchased from Sigma-Aldrich (St. Louis, MO, USA).

Tumor necrosis factor (TNF) -α was purchased from R&D systems (Minneapolis, MN, USA). It was reconstituted at 100 μg/ml in sterile PBS and stored at −80 °C; the TNF-α solution was diluted in serum-free medium to a concentration of 10 ng/ml when added to the cells. BAY 11–7082, Gö6976 and Sotrastaurin were purchased from Selleckchem (Houston, TX, USA). They were reconstituted in DMSO, and when added to the cells, 10 μL of DMSO was added per 1.0 ml of media as the control. Phorbol 12-myristate 13-acetate (PMA) was purchased from Sigma-Aldrich (St. Louis, MO, USA).

### Small interfering RNA, plasmids and cell transfections

To conduct the PKCα or p65 knockdown, three pairs of small interfering RNAs (siRNAs) against PKCα or p65 were purchased from GenePharma (Shanghai, China). Sequences of the siRNAs are listed in Additional file 1: Tables S2 and S3. To detect NF-κB activity, nucleotides of the NF-κB promoter were cloned into PGL3-Luc-vector, and the sequence was 5′-GGGAATTTCCGGGAATTTCCGGGAATTTCCGGG-AATTTCC-3′. The NF-κB luciferase plasmid was also purchased from GenePharma.

Cell transfection was performed using Lipofectamine™ 3000 (Invitrogen, Carlsbad, CA, USA) according to the manufacturer’s instructions. Briefly, the Lipofectamine™ 3000 reagent and RNA were separately diluted with Opti-MEM™ medium at room temperature and gently vortexed for 2–3 s. Then, the diluted RNA was added to the diluted Lipofectamine™ 3000 reagent and incubated for 5 min, and the RNA-lipid complex was added to the cells. The cell medium was replaced with complete medium after six hours, and the transfection efficiency was measured at 48 h post-transfection.

### TUNEL staining assay

Apoptotic DNA fragmentation was examined using a Cell-Light™ EdUTP TUNEL Cell Detection Kit (Ribobio, Guangzhou, Guangdong, China) according to the manufacturer’s protocol. Briefly, cells were seeded in 96-well plates and treated with DMSO, BAY 11–7082 (500 μM for 5637 and 200 μM for T24), or BAY 11–7082 combined with PMA (10 ng/ml) for 24 h. Cells were fixed with 4% paraformaldehyde at 4 °C for 30 min, permeabilized with 0.1% Triton X-100, and labeled with fluorescein-12-dUTP using terminal deoxynucleotidyl transferase. The localized red fluorescence of the apoptotic cells from fluorescein-12-dUTP was visualized using an inverted fluorescence microscope (Olympus, Tokyo, Japan) and captured under an original magnification of 400×. The apoptotic index was measured as the percentage of the terminal deoxynucleotidyl transferase–mediated dUTP nick end labeling (TUNEL)-positive cells.

### Cell apoptosis by flow cytometry

Cells (3 × 10^4^ per well) were seeded into 24-well culture plates and cultured for 24 h. Then, the cells were treated using the indicated reagents and methods for the indicated study purpose. The cells were harvested, washed three times in PBS, and resuspended in 0.4 ml of ice-cold PBS. The resuspended cells were incubated with propidium iodide (PI) and a fluorescein isothiocyanate (FITC)-conjugated monoclonal antibody specific for Annexin V (BD, San Diego, CA, USA). The results were measured by flow cytometry (Becton Dickinson Biosciences, San Jose, CA), and the data were analyzed using the ModFit LT software package. The experiments were performed independently in triplicate for each cell line.

### Dual luciferase reporter assays

Cells (3 × 10^4^ cells per well) were seeded in 24-well culture plates and allowed to settle. Then, cells were separately subjected to the indicated reagent treatment. The luciferase and Renilla signals were measured using a Dual Luciferase Reporter Assay Kit (Promega, Madison, WI, USA) according to the manufacturer’s protocol. The ratio of the firefly luciferase activity against the corresponding Renilla luciferase activity was used to conduct the statistical analysis. Each experiment was independently repeated three times.

### Immunofluorescence

To detect the nuclear trafficking of the NF-κB p65 subunit, the 5637, T24 and BIU-87 cells were seeded in 24-well plates and incubated with PMA (10 ng/ml) for 1 h at 37 °C. Cells were fixed with 4% paraformaldehyde for 30 min, permeabilized in 0.2% Triton X-100 for 30 min, washed with PBS, blocked with 1% BSA/0.05% Triton X-100 for 30 min, and further incubated with rabbit polyclonal antibody against NF-κB p65 (1:100 dilution) in blocking buffer overnight. The next day, cells were rewarmed at 37 °C for 1 h, washed with PBS, and incubated with anti-rabbit Alexa-Fluor 488 secondary antibody (Origene, Beijing, China) in blocking buffer for 60 min. After three washes in PBS, cells were incubated with DAPI (Beyotime, Shenzhen, Guangdong, China) diluted in PBS (10 ng/ml) for 20 min and washed with PBS three times. Immunofluorescence images were viewed using an inverted fluorescence microscope (Olympus, Tokyo, Japan) and captured under an original magnification of 400×.

### Immunohistochemistry

The expression of NF-κB p65 and PKCα in tumor tissues was detected using an UltraSensitive™ SP (Mouse/Rabbit) IHC kit (Maxin-Bio, Fuzhou, Fujian, China) according to the manufacturer’s instructions. Briefly, sections were dewaxed in xylene and ethanol. Antigen retrieval was performed using a microwave for 10 min at 100 °C. The sections were then incubated with rabbit anti-p65 or anti-PKCα antibody (1:200 dilution) (Abcam, Cambridge, MA, USA) for 1 h, followed by biotinylation with an anti-IgG antibody and streptavidin-biotinylated-complex horseradish peroxidase. For both antigens, DAB and hematoxylin were used for nuclear staining. The images were captured using an Upright Metallurgical Microscope (Olympus, Tokyo, Japan) under an original magnification of 400×.

### Statistical analysis

A statistical analysis was performed using SPSS (Statistical Package for the Social Sciences) 13.0 (SPSS Inc., Chicago, IL, USA). The results are presented as the mean ± SD unless otherwise stated. *P* < 0.05 was considered to indicate significant differences. A two-tailed Student’s t-test was used to assess significant differences between two groups of data in all pertinent experiments. Pearson’s correlation coefficient analysis was used to determine the correlation of expression between the genes.

## Results

### Expression profile of PKC isotypes and NF-κB p65 subunit in bladder cancer cell lines and tissue specimens

**Fig. 1:**
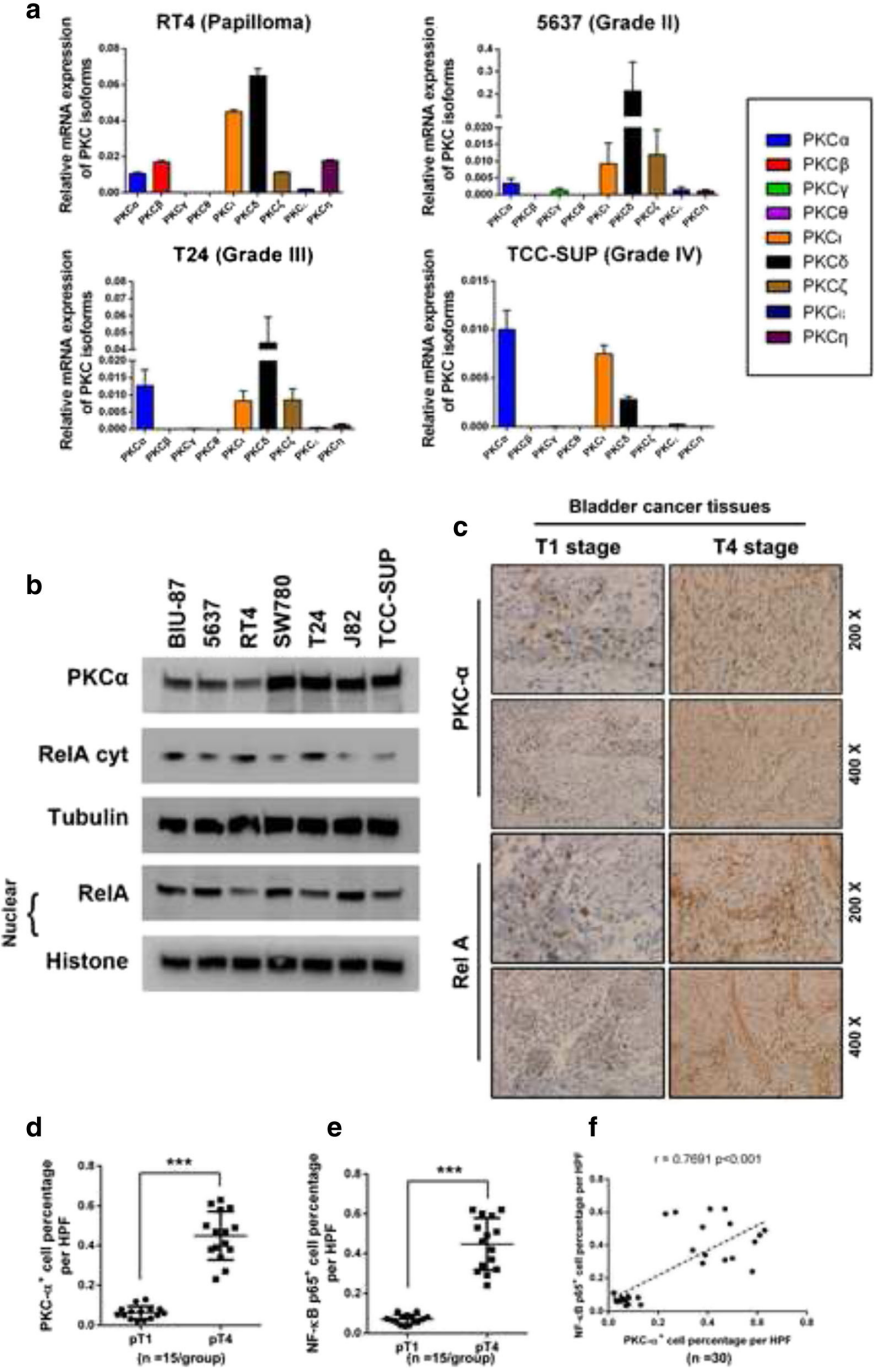
Expression profile of the PKC isotypes and NF-κB p65 subunit in bladder cancer cell lines and tissue specimens. **a** The expression profile of nine PKC isotypes in four bladder urothelial cancer cell lines was measured by real-time PCR. The expression levels were normalized to β-actin. The statistical analysis results are as follows. RT4 cell line (left upper panel): PKCδ, PKCι, PKCβ, PKCη vs PKCα: *p* < 0.01**; PKCζ vs PKCα: not significant. 5637 cell line (right upper panel): PKCδ, PKCι, PKCζ vs PKCα: *p* < 0.01**. T24 cell line (left lower panel): PKCδ vs PKCα: *p* < 0.01**; PKCα vs PKCι, PKCζ: *p* < 0.05*. TCC-SUP cell line (right lower panel): PKCα vs PKCι: not significant; PKCα vs PKCδ: *p* < 0.01**. **b** Protein expression of PKCα in seven bladder cancer cell lines was detected by western blot. The gels were run under the same experimental conditions. The band intensities were calculated using the ImageJ 1.46r software. β-Tubulin was used as an internal control for total protein measurements, and Histone was used as a nucleoprotein reference. The ratio of the target gene to β-Tubulin/Histone was used to conduct the statistical analysis. *P < 0.05 and **P < 0.01, as determined by Student’s T-test. **c** PKCα and NF-κB p65 expression were associated with tumor progression in 30 clinical bladder cancer specimens. Two representative cases are shown. The gene expression level was evaluated in three random visual fields. Original magnifications: 200× and 400×. The gene expression of PKCα and NF-κB p65 between tumor tissue samples staged as pT1 and pT4 was compared **d** and a Pearson’s correlation coefficient analysis was performed to analyze the expression correlation between the two genes

To investigate the expression pattern of PKCs in bladder cancer, we screened the mRNA expression of all PKC isotypes in four bladder cancer cell lines: RT4, 5637, T24 and TCC-SUP (Fig. 1a). The chosen cell lines were individually obtained from bladder cancer samples with increasing tumor stages of bladder papilloma, stage II, stage III, and stage IV. The results by real-time PCR showed that in the RT4 cell line (bladder papilloma), the mRNA expression of PKCα ranked sixth compared with the mRNA expression of other PKC isotypes (PKCδ, PKCι, PKCβ, PKCη vs PKCα: *p* < 0.01**; PKCζ vs PKCα: not significant). With the progression of tumor malignancy, expression of PKCα revealed a significant elevation compared with the other PKC isotypes: PKCα mRNA expression ranked fourth in 5637 (stage II), second in T24 (stage III) and first in TCC-SUP (stage IV). This result demonstrated that in the bladder cancer cell lines, within the isotypes of the PKC family, expression of PKCα revealed a strong tendency to be consequently increasing with the progression of tumor malignancy, indicating a critical regulatory role for PKCα in advanced bladder tumors. We further detected the protein expression of PKCα and the nuclear NF-κB subunit P65/RelA in seven bladder cancer cell lines by western blot (Fig. 1b), but no significant correlation in expression was found between the two genes. Next, we measured the expression of PKCα and NF-κB in bladder tumor tissues from 30 patients diagnosed with bladder cancers staged as pT1 (*n* = 15) and pT4 (*n* = 15) by immunostaining (Fig. 1c). We discovered that with the pathological progression of bladder cancer, the expression of these two genes revealed a remarkable elevation (Fig. 1d, e). Meanwhile, the Pearson’s correlation coefficient analysis revealed a significant correlation between the expression of PKCα and the NF-κB subunit (Fig. 1f). Taken together, these results suggested that PKCα was very likely to play a crucial role in bladder cancer tumorigenesis. Furthermore, the expression of PKCα and NF-κB was significantly correlated with the pathological progression of bladder cancer, and a positive expression correlation between the two genes was also confirmed in the cancer tissue specimens.

### PMA significantly induced overexpression of PKCα, p-PKCα and nuclear translocation of p65 in bladder cancer cell lines

**Fig. 2:**
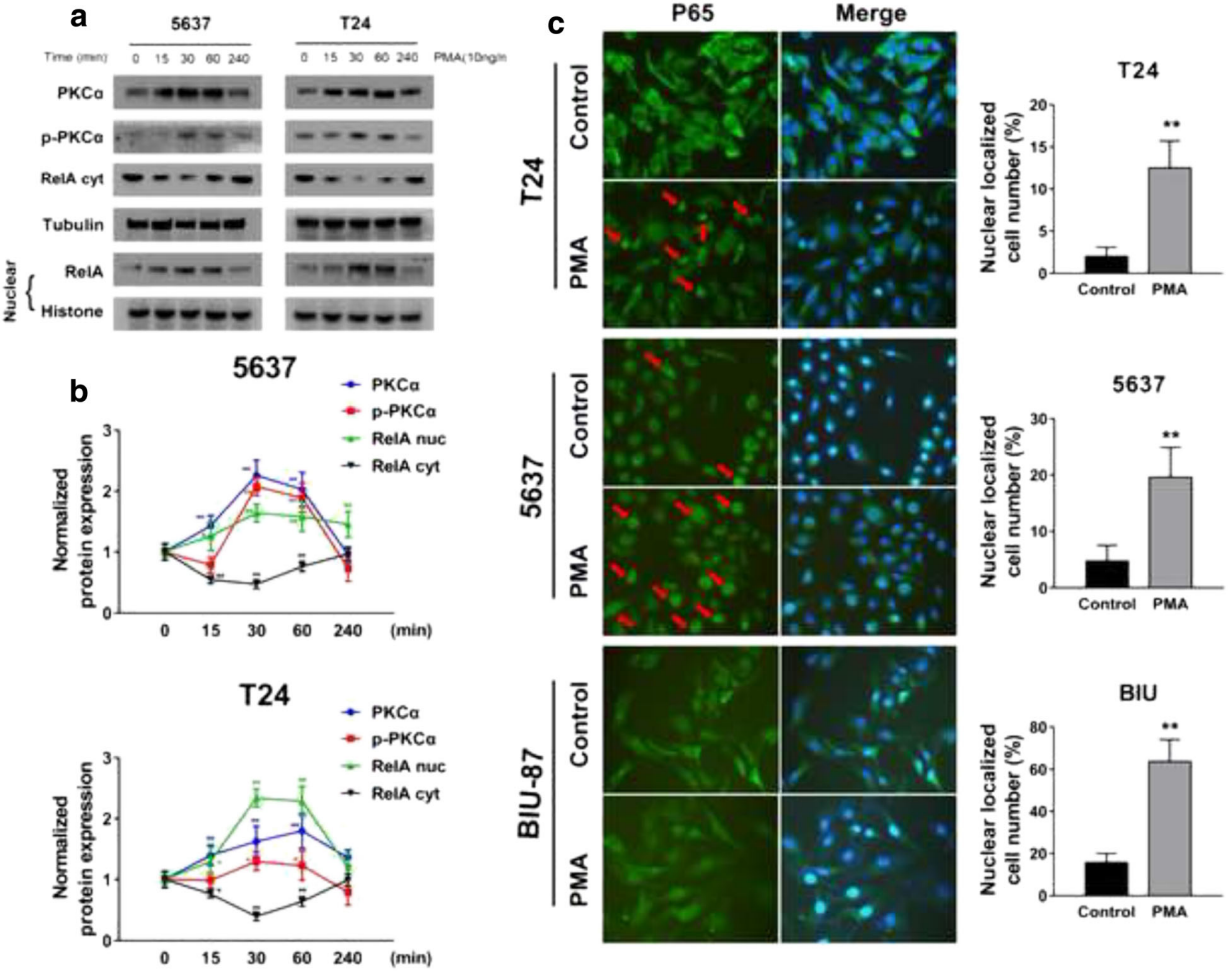
PMA significantly induces overexpression of PKCα, p-PKCα and NF-κB p65 nuclear translocation in bladder cancer cell lines. **a** 5637 and T24 cells were treated with PMA (10 ng/ml) for 0, 15, 30, 60, and 240 min, and the total, nuclear and cytoplasmic proteins were extracted at the indicated time point; p-PKCα and nuclear/cytoplasmic p65 were measured by western blot. **b** Normalized protein expression levels were calculated and analyzed. The gels were run under the same experimental conditions. The band intensities were calculated using the ImageJ 1.46r software. β-Tubulin was used as an internal control for the total protein measurement, and Histone was used as a nucleoprotein reference. The ratio of the target gene to β-Tubulin/Histone was used to conduct the statistical analysis. *P < 0.05 and **P < 0.01, as determined by Student’s T-test. **c** Cells were treated with DMSO or PMA (10 ng/ml) for 1 h, and p65 localization was detected by immunofluorescence. The cells with nuclear translocation of p65 are indicated with red arrows for the 5637 and T24 cell lines. For the BIU-87 cell line, nuclear translocation of p65 is evident in almost all cells within the visual field after the PMA treatment, and p65 expression can be observed in both the cytosol and nucleus. Original magnification: 400×. Comparisons between the control and PMA groups were made based on the statistical analysis of the cells with nuclear localization of p65 counted in three random fields

We asked whether the PKCs could actually activate NF-κB signaling in bladder cancer. We stimulated the expression of the PKCs with propylene glycol monomethyl ether acetate (PMA) in a time-dependent manner, and at the indicated time points, expression of PKCα, p-PKCα and nucleus NF-κB was measured by western blot. Figure 2a shows that PMA significantly induced the overexpression of PKCα and p-PKCα. Accordingly, nuclear expression of p65 was also increased, and the upregulation trend was generally consistent with the overexpression of PKCα (Fig. 2B). To confirm the result, we treated the tested cells with PMA (10 ng/ml) for 60 min, and the localization of the p65 protein was observed by immunofluorescence. To clarify the nuclear and cytoplasmic localization, another spindle-shaped bladder cancer cell line, BIU-87, was selected for the experiment. As Fig. 2c shows, after the PMA treatment, the number of cells with the nuclear localization of p65 were noticeably increased in all three cell lines. These results demonstrated that PMA stimulated the overexpression and phosphorylation of PKCα and induced the nuclear translocation of NF-κB p65.

### PKCα was the key player in PMA-induced NF-κB activation in bladder cancer

**Fig. 3:**
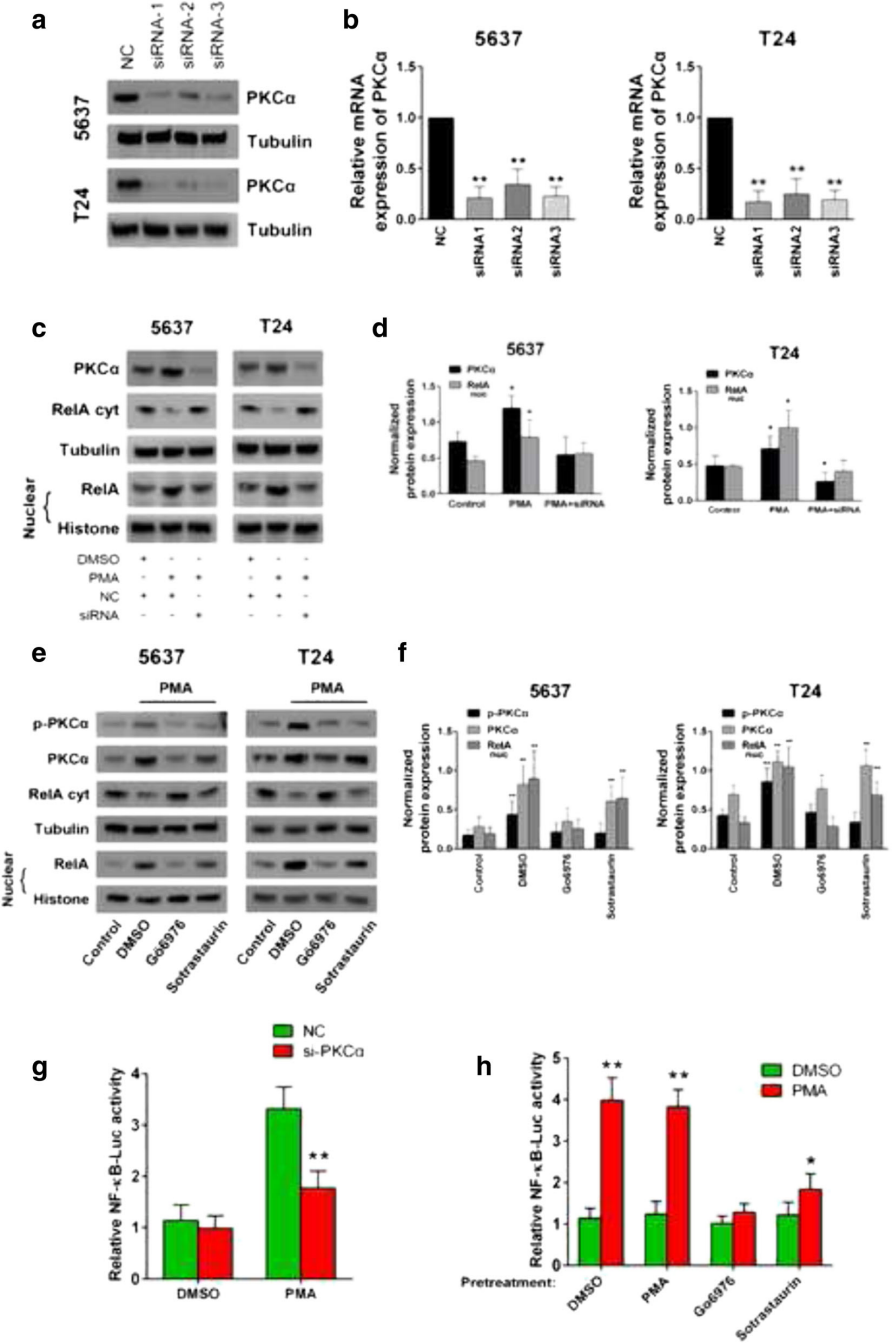
PKCα is the key player in PMA-induced NF-κB activation. Three pairs of small interfering RNA against PKCα were designed, and the knockdown efficiencies were analyzed by real-time PCR (**b**) and western blot (**a**). **c** Cells were treated/transfected with DMSO/negative control (NC), PMA/NC or PMA/siPKCα for 12 h, and protein expression of PKCα and nuclear/cytoplasmic p65 were detected by western blot. The experiment was repeated three times with each pair of siRNAs against PKCα, and similar results were obtained. A dual luciferasy reporter assay was performed in parallel to confirm the result (**g**). **d** Protein expression levels were normalized to Tubulin/Histone, and the band intensities were calculated and analyzed. (**e**) Cells were pretreated with DMSO, Gö6976 (100 nM) or Sotrastaurin (100 nM) for 1 h and then challenged with PMA (10 ng/ml) for 12 h. Cells without any treatment were used as the blank control. Protein expression of PKCα, p-PKCα and nuclear/cytoplasmic p65 were detected by western blot, normalized and analyzed against the internal control (**f**). Also a dual-luciferasy reporter assay was performed in parallel to confirm the reslut (**h**). The gels were run under the same experimental conditions. The band intensities were calculated using the ImageJ 1.46r software. β-Tubulin was used as an internal control for the total protein measurements, and Histone was used as a nucleoprotein reference. The ratio of the target gene to β-Tubulin/Histone was used to conduct the statistical analysis. *P < 0.05 and **P < 0.01, as determined by Student’s t-test

We confirmed that PMA was capable of inducing NF-κB nuclear translocation. Moreover, previous data suggested that PKCα was very likely to be the dominant functional isotype of the PKC family in advanced bladder cancer. Therefore, we next investigated whether PKCα was the key player in PMA-induced NF-κB activation. Three pairs of small interfering RNAs (siRNAs) were used to knock down the PKCα gene, and the knockdown efficiencies were confirmed by real-time PCR and western blot (Fig. 3a and b). Next, we treated the cells with PMA (10 ng/ml) for 60 min. As a comparison, we used another group of cells that were pre-transfected with the PKCα siRNA for 24 h followed by the same PMA treatment, and expression of PKCα and nuclear RelA was subsequently detected by western blotting. Figure 3c and d show that, as we had verified in previous data, PMA alone significantly enhanced the expression of PKCα and nuclear p65. In contrast, the stimulatory effect of PMA for p65 nuclear translocation could no longer be observed in the PKCα-knockdown cells. A similar result was also obtained in the NF-κB luciferase activity measurement (Fig. 3g).

We further confirmed the above result using two types of PKC inhibitors: a PKCα/β-specific inhibitor, Gö6976, and a general PKC inhibitor, Sotrastaurin. We separately pretreated the cells with DMSO, Gö6976 (100 nM) or Sotrastaurin (100 nM) for 1 h, after which the cells were challenged with PMA (10 ng/ml) for 12 h and subjected to a western blot analysis. Figure 3E and F show that compared with the control, the DMSO pretreatment and PMA noticeably stimulated the expression of PKCα, p-PKα and nuclear p65. In contrast, compared with the DMSO pretreatment, the two inhibitors dramatically inhibited PKCα, p-PKCα and nuclear p65 expression, which was otherwise elevated by PMA. Moreover, there was no significant difference in the reduction of nuclear p65 expression between the cells pretreated with Gö6976 and Sotrastaurin. This result demonstrated that general PKC and PKCα/β-specific inhibition had similar abilities toward inhibiting PMA-induced NF-κB nuclear translocation. A similar result was also obtained in the NF-κB luciferase activity measurement (Fig. 3h). Considering the extremely low mRNA expression of PKCβ (for the real-time PCR analysis, the Ct values for PKCβ were over 40 cycles in the 5637, T24 and TCC-SUP cell lines, Fig. 1A) in the bladder cancer cell lines, we concluded that PKCα played the dominant role in PMA-induced NF-κB activation.

### PKCα suppressed cells apoptosis by activating NF-κB signaling

**Fig. 4:**
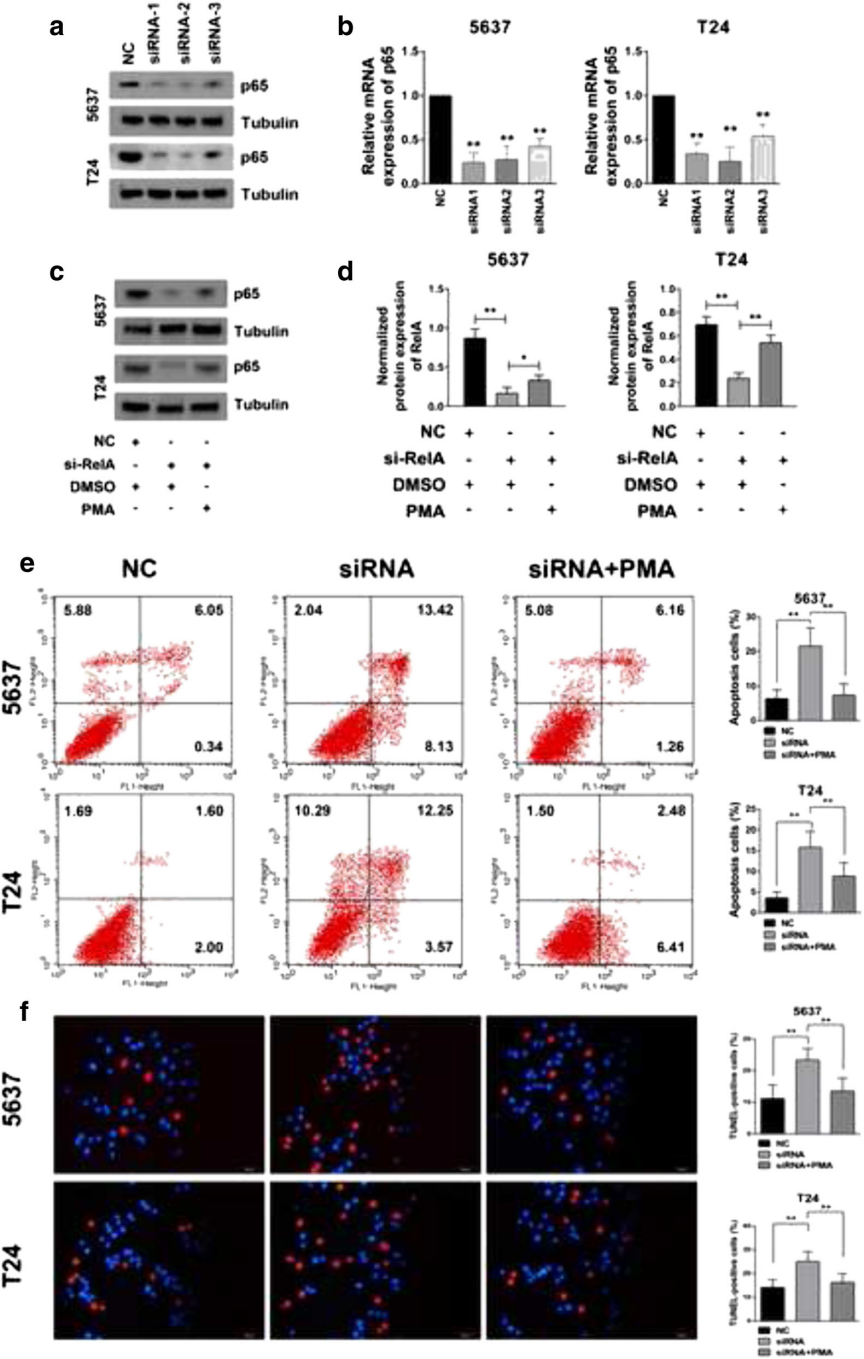
PKCα suppressed cell apoptosis by activating NF-κB signaling. Transfection efficiency of siRNA against p65 was measured by western blot **a** and real-time PCR **b**. **c**, **d** PMA restored the NF-κB expression that was effectively knocked down by the siRelA transfection. **e** Cells were transfected with the negative control (NC), p65 siRNA, or p65 siRNA plus the PMA (10 ng/ml) treatment for 24 h and then collected, stained with Annexin-V/PI and subjected to FACS for apoptotic analysis. The percentage of apoptotic cells (early and late apoptosis) were analyzed. **f** TUNEL staining was also performed to determine the cell apoptosis level. TUNEL-positive cells were counted and analyzed. Original magnification: 200×. *P < 0.05 and **P < 0.01, as determined by Student’s T-test. The experiment was repeated three times with each pair of siRNAs against p65, and similar results were obtained

As it has been firmly established that NF-κB signaling is closely related to cell cycle and apoptosis control, we further investigated whether the PKCα/NF-κB axis affected bladder cancer cellular function. Three pairs of siRNAs were designed and synthesized to silence the NF-κB p65 gene, and the knockdown efficiencies were confirmed by RT-PCR and western blotting (Fig. 4a and b). Then, we detected the alterations in cellular apoptosis after NF-κB p65 expression was inhibited or otherwise restored by PKCα. We separately transfected cells with the negative control oligo (NC), p65 siRNA, or p65 siRNA combined with a 24-h treatment with PMA (10 ng/ml). Cell apoptosis was then determined by both FACS and TUNEL staining. Figure 4E and F show that the p65 knockdown significantly induced cell apoptosis compared with the NCs. In contrast, PMA partially restored p65 expression (Fig. 4C and D) and significantly suppressed cell apoptosis, which was otherwise induced by the p65 knockdown alone. This result suggested that in bladder cancer, PKCα could potentially suppress cancer cell apoptosis by promoting NF-κB activation.

## Discussion

In the present study, our data demonstrated that within the PKC family, PKCα was very likely to play the dominant functional role in regulating NF-κB activity in bladder cancer. To confirm these results, we performed a series of tests on bladder cancer cell lines with pharmacological treatments, namely, PMA and PKC inhibitors Gö6976 and sotrastaurin, and measured p65 nuclear localization and NF-κB luciferase activity. Moreover, an NF-κB p65 gene knockdown was performed to induce cell apoptosis, whereas PMA was combined with the siRNA to suppressed cell apoptosis, which would otherwise be significantly induced by the p65 knockdown alone. We concluded that in bladder cancer, PKCα enhances cell resistance to apoptosis by stimulating NF-κB p65 nuclear translocation and that the PKCα/NF-κB cascade might play a crucial role in the tumorigenesis and progression of bladder cancer.

Since PKCs have been identified as the natural targets of phorbol esters, which possess tumor-promoting activity, studies examining the biological function of each individual isozyme, especially in carcinogenesis, have been going on for decades. Each PKC isotype may play a distinct role in regulating cellular function according to different cancer cell phenotypes and cell conditions. Unlike the nPKCs that are strictly expressed in certain tissues and cell types, PKCα is widely expressed in various tumor tissues. Previously, the involvement of PKCα in tumor promotion and progression was mainly discussed in gastrointestinal cancer, breast cancer and glioma. More recent studies have revealed the role of PKCα in bladder urothelial cell cancer. Our group has reported the novel function of PKCα in bladder cancer where it regulates cell survival through the netrin-1/UNC5B pathway [24], and by targeting DICER, PKCα can also modulate apoptosis of UCC cell lines [25]. In a study where a cohort of 56 pairs of bladder cancer tissue and adjacent normal tissue samples were analyzed, expression of PKCα and the ratio of PKCα expression in the nuclear membrane relative to the cytosol were found to be much higher in tumor tissues than in normal tissues [12]. These studies uncovered the mechanism underlying PKCα-mediated stimulation of malignant transformation of the bladder.

In this study, we detected the mRNA expression levels of all PKC isozymes in four bladder cancer cell lines that were obtained from bladder cancer tumor tissues staged as urothelial papilloma and II-IV. In addition, the result showed a notable elevation in PKCα expression that was consistent with the progression of the tumor, compared with the other PKC isotypes. We also measured the PKCα expression profile in seven bladder cancer cell lines, and a western blot analysis showed that PKCα expression revealed a tendency to be upregulated in advanced bladder cancer. IHC staining also confirmed the result. The above data were novel, identified the tumorigenic role of PKCα in bladder cancer, and provided solid evidence for further studies of the biological and carcinogenic functions of PKCs in bladder cancer. We also observed a very high expression level of PKCδ and PKCι, suggesting that they might also participate in the initiation of bladder cancer. Continuous investigations are still needed to study the protein expression pattern and the regulation mechanism. Here, we observed that PMA apparently stimulated nuclear NF-κB, while Gö6976 and sotrastaurin revealed similar abilities to inhibit PMA-induced NF-κB activation (Fig. 3E, H). This suggested that a specific PKC isoform, mainly the PKCα isoform, is responsible for the activation of NF-κB in bladder cancer, based on data using sotrastaurin, a general nonselective PKC inhibitor, versus Gö6976, which is more selective for PKCα/β [26]. Compared with the specific PKCα/β inhibitor, inhibition of general PKCs did not produce an extra NF-κB deactivation effect against PMA.

We also noticed that the protein expression correlation between PKCα and nuclear p65 was not significant when the cell lines were analyzed. This result is difficult to interpret because the activation of NF-κB is under the control of a wide variety of cellular signal networks in which PKCα is just one link in the processes. Previous studies have described hypotheses regarding the mechanisms of PKC-mediated regulation of NF-κB activation in several cell types and tumor models. In lymphocytes, PKC-θ activates NF-κB through phosphorylation and activation of the membrane-associated guanylate kinase (MAGUK) CARMA1 (also called Card11) [19]. In fibroblasts, PKCα stimulates NF-κB activation through downstream factor Raf-1. However, in bladder cancer, the underlying mechanism of PKC-mediated regulation of NF-κB activation and promotion of tumor progression still needs to be investigated.

## Conclusion

We have shown that in bladder cancer, PKC-α is highly expressed in progressed cancer tissues. Moreover, PKC-α enhances cell resistance to apoptosis and stimulates tumor malignancy progression by mediating NF-Kappa-B activation.

## Additional file

Additional file 1: Table S1. Primers used for Real time RT-PCR; **Table S2.** Sequence of small interfering RNA (siRNA) against PKCα; **Table S3.** Sequence of small interfering RNA (siRNA) against p65 (DOCX 28 kb)

## Abbreviations

BUCC: Bladder urothelial cell carcinoma
IKK: IκB kinase
MAGUK: Membrane associated guanylate kinase
NF-κB: Nuclear factor Kappa-B
PKC: Protein Kinase C
PMA: Phorbol 2-myristate 13-acetate
siRNA: small interfering RNA
UCCs: Urothelial cell carcinomas.
