# Supplementary material for: Protein kinase C-α (PKCα) modulates cell apoptosis by stimulating nuclear translocation of NF-kappa-B p65 in urothelial cell carcinoma of the bladder
Source: BMC Cancer. 2017 Jun 19;17:432. doi: 10.1186/s12885-017-3401-7 (PMC5477139; doi:10.1186/s12885-017-3401-7)
Supplement: Additional file 1: Table S1. — Primers used for Real time RT-PCR; Table S2. Sequence of small interfering RNA (siRNA) against PKCα; Table S3. Sequence of small interfering RNA (siRNA) against p65 (DOCX 28 kb) [file 12885_2017_3401_MOESM1_ESM.docx]

**TABLE S**

**Table S1. Primers used for Real time RT-PCR**

| Name | 5’-3’ sequence |
| --- | --- |
| PKC-α F | 5' GGAACCACAAGCAGTATT 3' |
| R | 5' GTCCTTCTGAATCCAACAT 3' |
| PKC-β F | 5' GGATTGGGATTTGACCAGCAG 3' |
| R | 5' TGGCACAGGCACATTGAAGT 3' |
| PKC-γ F | 5' AGCCACAAGTTCACCGCTC 3' |
| R | 5' GGACACTCGAAGGTCACAAAT 3' |
| PKC-δ F | 5' GTGCAGAAGAAGCCGACCAT 3' |
| R | 5' CCCGCATTAGCACAATCTGGA 3' |
| PKC-ε F | 5' CGAGGCCGTGAGCTTGAAG 3' |
| R | 5' GCAATGTAGGGGTCGAGAAGG 3' |
| PKC-ζ F | 5' AGAGCCTCCAGTAGACGACAA 3' |
| R | 5' CGGGATGAGGAAATGTAAGCAA 3' |
| PKC-η F | 5' CTGGACCCCTATCTGACGGT 3' |
| R | 5' TGTACGTGGGTTTGTTGGTCT 3' |
| PKC-θ F | 5' TGGACAATCCCTTTTACCCACG 3' |
| R | 5' GTCTCTGGAGGGGCAAGATTCA 3' |
| PKC-ι F | 5' AGGTCCGGGTGAAAGCCTA 3' |
| R | 5' TGAAGAGCTGTTCGTTGTCAAA 3' |
| β-actin F | 5' ACTTAGTTGCGTTACACCCTT 3' |
| R | 5' GTCACCTTCACCGTTCCA 3' |

F, forward; R, reverse

**Table S2. Sequence of small interfering RNA (siRNA) against PKCα**

| Name | 5’-3’ sequence |
| --- | --- |
| siRNA-1 sense | 5’ GCUCCACACUAAAUCCGCATT 3’ |
| antisense | 5’ UGCGGAUUUAGUGUGGAGCGG 3’ |
| siRNA-2 sense | 5’ UGCGGAUUUAGUGUGGAGCGG 3’ |
| antisense | 5’ UUCCGGAAUGGGUACGUUGTA 3’ |
| siRNA-3 sense | 5’ GGCUGUACUUCGUCAUGGATT 3’ |
| antisense | 5’ UCCAUGACGAAGUACAGCCGA 3’ |

F, forward; R, reverse

**Table S3. Sequence of small interfering RNA (siRNA) against p65**

| Name | 5’-3’ sequence |
| --- | --- |
| siRNA-1 sense | 5’ GGAGUACCCUGAGGCUAUATT 3’ |
| antisense | 5’ UAUAGCCUCAGGGUACUCCAT 3’ |
| siRNA-2 sense | 5’ CCCUUUACGUCAUCCCUGATT 3’ |
| antisense | 5’ UCAGGGAUGACGUAAAGGGAT 3’ |
| siRNA-3 sense | 5’ GGACAUAUGAGACCUUCAATT 3’ |
| antisense | 5’ UUGAAGGUCUCAUAUGUCCTT 3’ |

F, forward; R, reverse
